# METTL9 mediated N1-histidine methylation of zinc transporters is required for tumor growth

**DOI:** 10.1007/s13238-021-00857-4

**Published:** 2021-07-04

**Authors:** Mengyue Lv, Dan Cao, Liwen Zhang, Chi Hu, Shukai Li, Panrui Zhang, Lianbang Zhu, Xiao Yi, Chaoliang Li, Alin Yang, Zhentao Yang, Yi Zhu, Kaiguang Zhang, Wen Pan

**Affiliations:** 1 Department of Gastroenterology, The First Affiliated Hospital of USTC, Division of Life Sciences and Medicine, University of Science and Technology of China, Hefei, 230026 China; 2 Hefei National Laboratory for Physical Sciences at Microscale, the CAS Key Laboratory of Innate Immunity and Chronic Disease, School of Basic Medical Sciences, Division of Life Sciences and Medicine, University of Science and Technology of China, Hefei, 230026 China; 3 Key Laboratory of Structural Biology of Zhejiang Province, School of Life Sciences, Westlake University, Hangzhou, 310024 China; 4 Institute of Basic Medical Sciences, Westlake Institute for Advanced Study, Hangzhou, 310024 China

**Dear Editor,**

Histidine methylation has been known for many years (Searle and Westall, 1951), but only a few proteins carrying such modifications have been studied (Webb et al., 2010; Al-Hadid et al., 2014; Kwiatkowski et al., 2018; Guo et al., 2019; Wilkinson et al., 2019; Kwiatkowski and Drozak, 2020). Recent proteomic studies suggest that more than 13% of all protein methylation events in human methylome was attributed to the modification of protein histidine residues (Ning et al., 2016). Histidine can be methylated at either N1 or N3 position of its imidazole ring, yielding the isomers 1-methylhistidine (His(1-me)) or 3-methylhistidine (His(3-me)). So far, only a limited number of histidine methyltransferases have been reported. Hpm1 and SETD3 modify N3-methylhisitidine on specific substrates, Rpl3 and actin, respectively (Webb et al., 2010; Wilkinson et al., 2019). CARNMT1 is an N1 position-specific methyltransferase that catalyzes dipeptide (Cao et al., 2018). However, the methyltransferases that modify N1-methylhisitidine on protein substrates, as well as the functional and (patho)physiological significance of such modification, still remain a major knowledge gap in the field of protein posttranslational modification.

Recent findings highlight methyltransferase-like protein family (METTL) as an important family of putative methyltransferase (Jiang et al., 2021). As *Mettl9* is broadly expressed in various cancer cell lines and its biological functions are unknown, we set out to study *Mettl9*. The targeting strategy of knocked-out *Mettl9* and the gene knockout efficiency were evaluated (Fig. S1A–C). RM-1 cells lack of *Mettl9* (hereafter termed *Mettl9* KO cells) showed significantly reduced growth and colony formation *in vitro* (Fig. 1A–C). Similar results were observed in *Mettl9* KO MC38 cells (Fig. S1D). Next, we found that deletion of *Mettl9* significantly inhibited tumor autonomous growth *in vivo* (Fig. 1D–F). Consistent with what we observed in immunodeficient mice, *Mettl9* ablation similarly resulted in compromised tumor growth in immunocompetent mice (Fig. 1G–I). Analysis of intratumoral immune cells showed that both the numbers and percentages of CD45^+^ cells were significantly increased in *Mettl9* KO tumors compared to WT tumors (Fig. S1E and S1F). Among intratumoral immune cells, the percentages of CD4^+^ and CD8^+^ effector T cells were significantly increased (Fig. S1G and S1H). Collectively, these results indicated that *Mettl9* deletion not only suppresses tumor cell autonomous growth, but also elicits potent anti-tumor immunity to restrain tumor burden.

**Figure 1:**
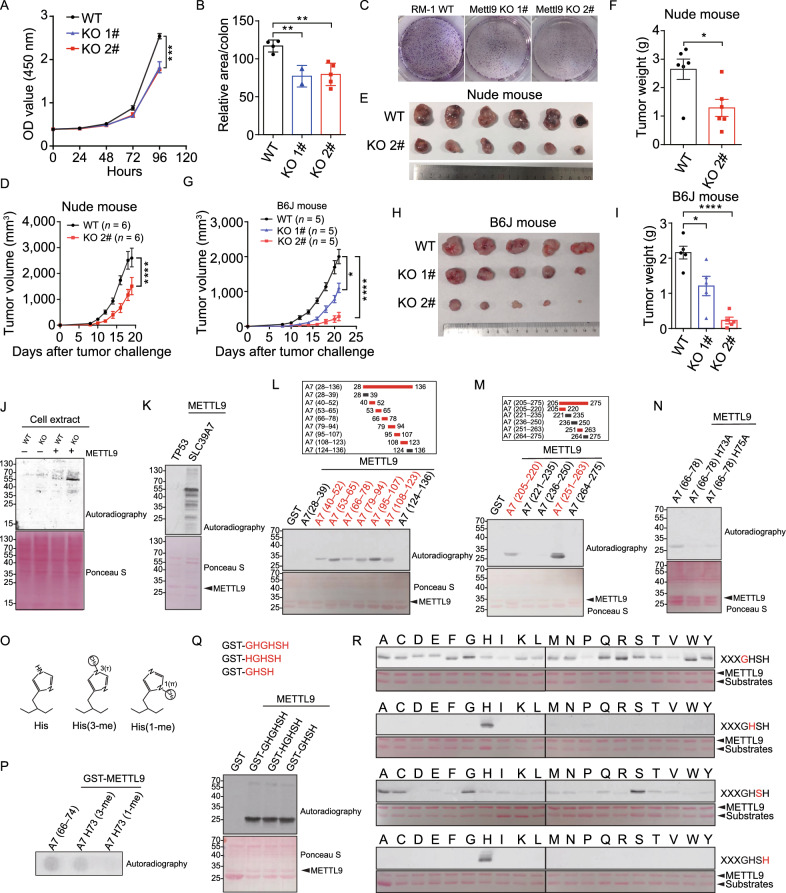
**METTL9 is a protein N1-histidine methyltransferase that is required for cell proliferation and tumor growth**. (A–C) Knockout of *Mettl9* in RM-1 tumor cells decreases cell growth. (A) Cell growth of wildtype (WT) and two different knockout (KO) clones of RM-1 cells was measured by CCK8 and (B and C) colony formation assay. (D–I) WT and *Mettl9* KO RM-1 tumor cells were injected into nude mouse and C57B6/J mice. (D and G) Tumor growth curves in nude mice (*n* = 6) and C57B6/J mice (*n* = 5). (E and H) Pictures of tumors three weeks after tumor cell injection. (F and I) Tumor weights for (E and H). (J–N) Enzymatically active METTL9 methylates SLC39A7. (J) Fluorography showing the activity of recombinant METTL9 on cell extracts from WT and *Mettl9* KO RM-1 cells in the presence of [^3^H]AdoMet. Ponceau S staining for total protein was used as loading control (bottom). (K) Fluorography showing recombinant GST-SLC39A7 was methylated by recombinant METTL9 in the presence of [^3^H]AdoMet. GST-P53 was used as a substrate control. (L and M) Fine mapping of the METTL9-methylated regions in SLC39A7. The methylated truncate was colored in red. (N) Fluorography showing *in vitro* activity of METTL9 on WT and mutated recombinant GST-A7 (66–78). (O) Histidine (His, left), 3-(τ)-methyl histidine (His(3-me), center); 1-(π)-methyl histidine (His(1-me), right). (P) METTL9 generates His73(1-me). *In vitro* methylation reactions with GST-METTL9 on the indicated peptides visualized by autoradiography. (Q) Fluorography showing GHSH motif is adequate for METTL9 mediated methylation. *In vitro* methylation assay of METTL9 on recombinant GST-GHGHSH, GST-HGHSH and GST-GHSH. (R) *In vitro* activity of METTL9 on recombinant protein arrays. Residue in the GHSH motif (red) was replaced with 20 different amino acids. For all panels, *P < 0.05; **P < 0.01; ***P < 0.001; ****P < 0.0001. Error bars represent S.D. Data are representative of three independent experiments

As we know, METTL9 belongs to the seven-β-strand (7BS) methyltransferase, characterized by a twisted beta-sheet structure and certain conserved sequence motifs (Petrossian and Clarke, 2011). We aligned the sequence of METTL9 in different species, and found that METTL9 has conserved sequence motifs that are similar to METTL family members that are responsible for protein methylation (Fig. S2A). We first demonstrated that purified recombinant mouse METTL9 protein could not methylate a histone substrate (Fig. S3A). Considering the fact that METTL9 protein was localized in the cytoplasm (Fig. S3B), we detected the activity of METTL9 to methylate proteins in WT and *Mettl9* KO tumor cell lysates and observed a substrate protein approximately 55-kDa was methylated (Fig. 1J), indicating that METTL9 indeed has enzymatic activity and is a non-histone protein methyltransferase. We next constructed *Mettl9*-3×flag knock-in RM-1 cell line and identified several interactants of endogenous METTL9 in tumor cells (Fig. S3C). Among them, SLC39A7 is a previously published METTL9 interactant (Ignatova et al., 2019), which is a zinc transporter, nearly 55KD and abundantly expressed in cell lysates (Fig. S3D). We next confirmed SLC39A7 was indeed an *in vitro* substrate of METTL9 (Fig. 1K). To fine-mapping of the methylation sites at SLC39A7, we performed *in vitro* METTL9 methylation assay on multiple truncated peptides (Figs. 1L and 1M, S3E and S3F). Two of them that could be methylated by METTL9 were further identified by mass spectrum to pinpoint the methylated residues. The results showed only histidine residues in these peptides were methylated (Fig. S3G and S3H). We observed that replacement of histidine by alanine abolished METTL9 mediated methylation (Fig. 1N). Correspondingly, although METTL9 could not methylate histone itself, it could indeed methylate histone H3 with a 6× His tag (Fig. S4A). As we know, histidine can potentially be methylated on the nitrogen in position 1 (π, N1) or 3 (τ, N3) of the imidazole ring, and endogenous SLC39A7 was identified carrying physiological methylation on histidine at N1 position (Wilkinson et al., 2019). We next performed *in vitro* methylation assays on a peptide (residues 66–74 of SLC39A7) that spanned His73 and in which His73 was either unmethylated or methylated at the N1 or N3 position (Fig. 1O), and observed that METTL9 methylated the unmodified peptide and the His73(3-me)-containing peptide, but not the His73(1-me)-containing peptide, indicating that METTL9 catalyzes methylation of His73 at the physiologically relevant N1 position (Fig. 1P). We also mutated several residues in METTL9 potential active sites, motif I and motif II regions and defined some key residues that are essential for sustaining METTL9 mediated histidine methylation (Fig. S4B). Collectively, all the above data suggest METTL9 is a protein N1 histidine methyltransferase.

We found that the above fine-mapped methylated short peptides of SLC39A7 all carry a common Gly-His-x-His-x-His (GHxHxH) sequence motif, most of them are GHGHSH. We further defined that a motif with four amino acid (GHSH) is adequate for METTL9 mediated methylation (Fig. 1Q). Next, we used GHSH as a base motif, substituted each residue for 20 different amino acids and generated a total 80 GST-tagged recombinant proteins. We observed that both the second and the fourth histidine of the motif were indispensable for histidine methylation *in vitro*, and alteration of either totally abolished METTL9 mediated methylation. The amino acid in the third x was preferably Alanine, Cysteine, Glycine, or Serine (ACGS), implying that small and uncharged residues in the middle were necessary for efficient histidine methylation. In addition, when the first amino acid was substituted by Isoleucine, Proline or Valine (IPV), METTL9 mediated histidine modification was significantly reduced (Fig.1R). These data suggest that METTL9 catalyzes the histidine methylation on a motif xHxH, where the first x is preferably not IPV and the second x is preferably ACGS. Besides, substrate proteins such as S100A9 and NDUFB3, with previously reported N1-methylhisitidine modification located on xHxH, could indeed be methylated by METTL9 (Fig. S5A). We also overexpressed HA-tagged full-length SLC39A7 in 293T cells, followed by pull-down and LS-MS analysis, and confirmed methylhistidine modifications of SLC39A7 *in vivo* (Fig. S5B). These results extended METTL9-mediated methylation on xHxH motif to several physiologically relevant substrates *in vivo*.

We next searched in the mouse proteome using METTL9 preferable recognition motif sequence (GHSH, GHAH, GHCH, GHGH), the results showed that the top hits were mainly enriched in zinc transporter families SLC39s and SLC30s (Fig. S5C). Like SLC39A7, most of zinc transporter members contain more than one xHxH motif in their sequences (Fig. S5D), implying that METTL9 might be critical for regulating zinc transporters and affecting cellular zinc concentration. To investigate whether METTL9 regulates cellular zinc concentrations, we utilized a fluorescent zinc probe (Fluozin-3) to evaluate the free zinc concentration in WT and *Mettl9* KO RM-1 tumor cell lines. In *Mettl9* KO cells, we observed that zinc level was significantly increased and aggregated in cytoplasm (Fig. 2A). The data support that *Mettl9* indeed plays an important role in regulating of zinc homeostasis in cells.

**Figure 2:**
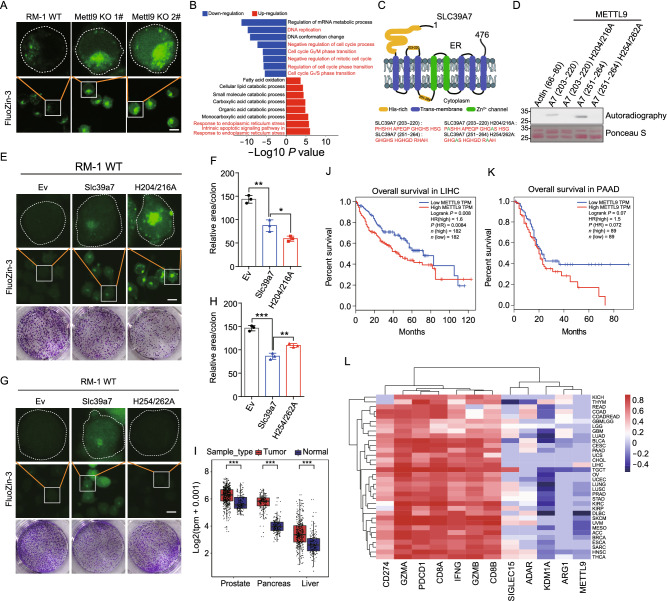
**METTL9 catalyzes methylhistidine residues of zinc transporter SLC39A7 and regulates cytoplasmic zinc homeostasis and cell proliferation**. (A) Fluorescence images of FluoZin-3 (2 μmol/L, 1 h, 25 °C) stained WT RM-1 cell line (left), *Mettl9* KO 1# cell line (middle), *Mettl9* KO 2# cell line (right). Scale bar, 10 μm. (B) GO analysis of top pathways from differentially expressed genes in WT and *Mettl9* KO RM-1 tumor cells. (C) The predicted structure model of SLC39A7. The number represents the peptide region in SLC39A7. Green color represents mutant residues. (D) *In vitro* activity of METTL9 on recombinant GST tagged WT and mutant peptides at the indicated region of SLC39A7. H204/216A, histidine to alanine mutation at 204 and 216 sites; H254/262A, histidine to alanine mutation at 254 and 262 sites. (E–H) Overexpression of WT and mutant SLC39A7 in RM-1 WT cells. (E and G) Fluorescence images of FluoZin-3 (2 μmol/L, 1 h, 25 °C) stained the indicated groups of BFP-empty vector (EV), BFP-SLC39A7, BFP-SLC39A7 H204/216A and H254/262A mutants. Scale bar, 10 μm. (F and H) Quantification of colony formation assays in (E and G) (see Methods). (I) *METTL9* transcript levels were analyzed in cancer and normal tissues from the TCGA database combined with GTEx normal data. PRAD (Prostate adenocarcinoma), PAAD (Pancreatic adenocarcinoma) and LIHC (Liver hepatocellular carcinoma). (J and K) The overall survival rates of the *METTL9* high-expression group and the *METTL9* low-expression group in PAAD and LIHC cancer types from the TCGA data. (L) Heatmap correlation between several genes expression levels and immune scores calculated by ESTIMATE in tumor tissues among different TCGA cancer datasets. For all panels, *P < 0.05; **P < 0.01; ***P < 0.001; unpaired two-tailed Student’s *t*-test. Error bars represent S.D. Data are representative of three independent experiments

As we know, abnormal zinc transport from ER to cytoplasm induces ER stress, and impaired cell proliferation (Adulcikas et al., 2018). We next performed RNA-seq analysis on WT and *Mettl9* KO RM-1 cells to evaluate the molecular downstream of *Mettl9*. As expected, Gene ontology (GO) enrichment analysis revealed that genes with reduced expression in *Mettl9* KO cells were significantly enriched in GO terms related to “Cell cycle” “DNA replication”, whereas those with increased expression in *Mettl9* KO cells were significantly enriched in “Endoplasmic Reticulum Stress” (Fig. 2B). We reanalyzed the published RNA-seq data from WT and *Slc39a7* Knock-down (KD) cells, and GSEA analysis showed that gene sets, such as “Cell cycle” or “DNA replication”, “Homologous recombination” and “Ribosome biogenesis in eukaryotes”, were commonly downregulated in both *Mettl9* KO and *Slc39a7* KD cells, compared to their corresponding controls (Fig. S6A–H). The data imply that, although METTL9 possibly modifies multiple zinc transporters, SLC39A7 appears to be an essential one of them mediating METTL9 downstream function in cells.

According to the previous studies, SLC39A7 harbors highly-conserved histidine-rich regions that potentially contribute to zinc binding, storage and release (Adulcikas et al., 2018; Zhang et al., 2019), although their exact functions are still unclear. We selected two METTL9 motif-containing histidine-rich regions (residues 203–220 and residues 251–264 of SLC39A7) for further investigation (Fig. 2C). We first confirmed that these two SLC39A7 peptides could be methylated by METTL9, whereas peptides with His to Ala mutation impaired METTL9 mediated methylation (Fig. 2D). We constructed two mutated constructs of full-length *Slc39a7*, each one replacing two targeted His residues to Ala to mimic the unmethylated status. We found that H204/216A mutation significantly upregulated zinc aggregation in cytoplasm compared to WT construct controls (Fig. 2E), while H254/262A mutation slightly downregulated zinc level (Fig. 2G). Consistent with the intracellular zinc levels, we observed that H204/216A mutation significantly downregulated colony formation (Fig. 2E and 2F), while H254/262A mutant slightly upregulated colony formation compared to SLC39A7 WT controls (Fig. 2G and 2H). Besides, we also mutated histidine “H” to the bulkier phenylalanine “F” to mimic the effect of methylation, although not perfect. We observed that H256F overexpression in *Mettl9* KO cells significantly downregulated zinc levels in cells, while H206/218F overexpression upregulated zinc levels accumulated (Fig. S7A). Although we have no clues to explain the complexity due to the lack of detailed structure information of SLC39A7, these results imply that methylation of different key residues in the outer and inner ER xHxH motifs may play unique roles in zinc storage and release.

We further evaluated *METTL9* expression in human tumor samples and found that *its* mRNA expression was significantly increased in some cancers compared with normal tissues, such as pancreatic cancer, liver cancer, and prostate cancer (Fig. 2I). In pancreatic cancer and liver cancer, higher expression of *METTL9* was associated with worse clinical outcomes (Fig. 2J and 2K). Moreover, we calculated the immune score of different cancer samples from TCGA database, and found that *METTL9* gene expression level negatively correlated with immune scores in most cancers (Fig. 2L).

Despite being identified for 70 years; histidine methylation has never been seriously studied (Searle and Westall, 1951). Recent proteomic studies suggest that the protein histidine methylation is a widespread modification in mammalians (Ning et al., 2016). However, it is largely unknown about the functional and (patho)physiological significance of such modification in cells and organisms, as well as the responsible methyltransferases (Kwiatkowski and Drozak, 2020). Here, by using a combination of *in vitro* and *in vivo* approaches, we identified METTL9 as a histidine methyltransferase that specifically catalyzes N1-methylhisitidine formation in distinct protein substrates. We demonstrated that METTL9 is required for tumor growth in cellular and animal models.

During the submission process of our manuscript, Shinkai and Falnes groups first reported METTL9 as a N1 histidine methyltransferase which methylates His-x-His motif in numerous substrates (Davydova et al., 2021). Their work also provides evidence for METTL9 mediated His(1-me) formation *in vivo*. Our work independently confirmed that METTL9 is a protein N1 histidine methyltransferase and established that METTL9 recognized an xHxH motif in the substrate protein. Our work also provides insights into the roles of METTL9 in tumorigenesis, and suggests its mediated methylation is of regulatory importance. Identifying selective and potent small-molecule inhibitors of METTL9 may thus represent a potential therapeutic strategy for anti-proliferative cancer drugs.

## Electronic supplementary material

Below is the link to the electronic supplementary material.

Supplementary material 1 (PDF 7564 kb)
