# Supplementary material for: METTL9 mediated N1-histidine methylation of zinc transporters is required for tumor growth
Source: Protein Cell. 2021 Jul 4;12(12):965–70. doi: 10.1007/s13238-021-00857-4 (PMC8674392; doi:10.1007/s13238-021-00857-4)

Supplemental figure legends

Figure S1. *Mettl9* is required for cell proliferation and tumor growth.

(A) The scheme of constructing *Mettl9* knockout cell line. CRISPR/Cas9 is used to remove exon 2 and 3 and exon 4 of *Mettl9*. **(B, C)** Validation of the efficiency of *Mettl9* knockout by measurement of mRNA and protein expression. **(D)** Knockout of *Mettl9* in MC38 tumor cells decreases cell growth measured by CCK-8 assay. **(E-H)** WT and *Mettl9* KO RM-1 tumor cells were injected into C57B6/J mouse (n=5). Tumor infiltrating immune cells from WT and *Mettl9* KO tumor-bearing C57B6/J mice were examined by flow cytometry. **(E, F)** The cell numbers per tumor weight and the percentages of tumor-infiltrating $CD45^+$ cells within all live cells from the tumor were quantified. Each dot represents one mouse. n=5. **(G, H)** Tumor-infiltrating $CD4^+$ and $CD8^+$ T cells were quantified as the percentages within total $CD45^+$ cells. Each dot represents one mouse. n=5. For all panels, *: $P< 0.05$; **: $P < 0.01$; ***: $P < 0.001$; ****: $P < 0.0001$. Error bars represent S.D. Data are representative of three independent experiments.

Figure S2. METTL9 is a conservative 7-beta Strand methyltransferase.

(A) Alignment of the core methyltransferase domain of METTL9 orthologues. Homo Sapiens (Hs; NP_057109.3), Mus musculus (Mm; NP_067529.2), Thamnophis elegans (Te; XP_032087096.1), Xenopus tropicalis (Xt; NP_001007899.1), Danio rerio (Dr; NP_001070810.1), Ostreococcus tauri (Ot; XP_003078360.1). Blue star, mutant at predicted motifs. Red star, mutant at predicted active sites.

Figure S3. METTL9 methylates Zn^{2+} transporter SLC39A7.

(A) Fluorography showing histone was not methylated by METTL9. GST-SDG714C as a positive control. **(B)** Immunofluorescence localization analysis of *Mettl9*-GFP fusion protein in B16F10 cell (exogenous) and *Mettl9*-flag fusion protein in RM-1 *Mettl9*-3×flag knock-in cells (endogenous). Scale bar, 10 μm. **(C)** The scheme of constructing flag-tag knock-in RM-1 tumor cell line and the experimental workflow. **(D)** The METTL9 binding proteins. Dotplot of METTL9-interacting proteins by immunoprecipitation–mass spectrometry (IP–MS) analysis. Colored indicates enriched proteins compared to control (PSMs > 2; log2(Sum PEP Score) > 2). **(E, F)** Fine mapping of the METTL9-methylated regions in SLC39A7. The diagram shows different recombinant GST-SLC39A7 (Termed A7) truncates. The methylated peptide was colored in red. **(G, H)** LC-MS/MS fragmentation spectra analysis of METTL9 methylated peptide SLC39A7(66-78) and SLC39A7(95-107). The monomethylation residues at His73, His75, His101 and His103 were colored in green.

Figure S4. The key sites of METTL9 enzymatic activity

(A) Fluorography showing recombinant histone 3 with 6×His tag was methylated by METTL9. GST-SDG714C as a positive control. Ponceau S-stained membrane as loading control (bottom). (**B)** *In vitro* activity of WT and mutated METTL9 on recombinant 6×His-histone H3. D151A, G153R, G155R, in motif-I; D204A, in motif-II; L175A, I180A, G266R, predicted active sites. The data are representative of three independent experiments

Figure S5. METTL9 methylates xHxH motif on zinc transporters.

(A) *In vitro* activity of METTL9 on recombinant GST-S100A9, GST-ZnT2 and GST-NDUB3 with GHxH motif. GST as a control substrate. **(B)** LC-MS/MS fragmentation spectra analysis of HA-SLC39A7 overexpressed in cells. The monomethylation residue at His256 was colored in green. **(C)** GO analysis of top pathways from proteins enriched with GHA/C/G/SH motif. **(D)** Evolutionary tree enrichment of proteins containing GHA/C/G/SH motif of ZnT (SLC30s) and ZIP (SLC39s) family.

Figure S6. *Mettl9* and *Slc39a7* regulates common downstream pathways

(A-H) Gene set enrichment analysis (GSEA) of differentially expressed genes in *Mettl9* KO cell or *Slc39a7* KD cells. Significant KEGG pathways with an enrichment score > 0.4 and p-value <0.01 were identified. Transcripts downregulated in *Mettl9* KO cell or *Slc39a7* KD cell versus their controls significantly enriched in **(A, E)** Cell cycle, **(B, F)** DNA replication, **(C, G)** Homologous recombination and **(D, H)** Ribosome biogenesis in eukaryotes.

Figure S7. SLC39A7 mutants affect the zinc level in *Mettl9* KO cells.

(A) Fluorescence images of FluoZin-3 (2 µM, 1 h, 25 °C) stained the indicated groups of BFP-empty vector (EV), BFP-SLC39A7, BFP-SLC39A7 H206/218F mutants, BFP-SLC39A7 H256F mutants. Scale bar,10 µm.

Supplemental Methods

Cell culture.

The RM-1, MC38, HEK239T cell line was purchased from American Type Culture Collection (ATCC, Manassas, VA, USA). All cell lines were cultured in high-glucose Dulbecco's modified Eagle's medium (H-DMEM: Gibco) supplemented with 10% fetal bovine serum (FBS; Gibco), 100 U/mL penicillin, and 100 ug/ml streptomycin. All cell lines were cultured at 37 °C with 5% CO2.

Plasmid construction and Peptides.

Slc39a7 (ZIP7) and its fragments and mutants were cloned into pGEX-6P-1. *Slc39a7* and its mutants were inserted into Plvx-IRES-BFP. *Slc39a7* were inserted into pcDNA 3.2 - 3'HA. *Mettl9* and its mutants were cloned into the pGEX-6P-1. *Mettl9* were inserted in pEGFP-N3. All the peptides were designed and inserted into pGEX-6P-1 by annealing. *S100a9*, *ZnT2* and *Ndub3* were cloned and inserted into pGEX-6P-1. 6×His Histone H3 was a gift from Yong Ding lab in USTC.

The following peptides were synthesized：SLC39A7(66-78): DFHHGHGHTHESI; SLC39A7(95-107): LHHGHSHGHSHDS; SLC39A7(66-74): DFHHGHGHT; SLC39A7(66-74) H73(3-me): DFHHGHG(His(3-me))T; SLC39A7(66-74) H73(1-me): DFHHGHG(His(1-me))T.

Generation of mouse *Mettl9* KO/KI cell lines.

The *Mettl9* gene was disrupted by the CRISPR-Cas9 method using four guide RNA targeted to introns of upstream (sgRNA1: 5′-AAAGATGATGATCGGCCTCA-3′; sgRNA2: 5′-GATGATGATCGGCCTCAGGG-3′) of exon 2 and downstream (sgRNA3: 5′-AGCCATGTGTAGTATCACCG-3′; sgRNA4: 5′-TACAGCATTCTGTACGCCCC-3′) of exon 4 in *Mettl9* were cloned into HP180 vector (A gift from Hui Yang lab). For the generation of RM-1 knock-in (KI) cells complemented with C-terminally 3×flag-tagged *Mettl9*, plasmids for *Mettl9*-flag donor (pUC19-*Mettl9*-3×flag) and guide RNA plasmids (sgRNA5: 5′-CTCAGACCAGTATAAACACG-3′; sgRNA6: 5′-TCTTCTGGAGGGTCGGTTGC-3′) were co-transfected into cells. Two days after the transfection, GFP positive cells were sorted and single cells were seeded into 96 well plate. The clones were screened by genomic PCR. The flag KI clones were screened by genomic

PCR and confirmed by Western Blot using anti-flag antibody.

Western Blots and antibodies.

Protein was extracted from the cells with IP buffer (25 mM Tris-HCl pH 7.4, 150 mM NaCl, 1 mM EDTA, 1% NP-40, 5% glycerol) and resolved on SDS–PAGE gels, then transferred to PVDF membranes. The primary antibodies against METTL9 (1:2000, ProteinTech) and β-actin (1:5000, ProteinTech) were used overnight at 4 ℃, after washing the membranes was incubated with secondary antibody (1:5000, ProteinTech) for 40 minutes. The membrane was explored by using enhanced chemiluminescence kit (Biosharp) according to the manufacturer's instructions.

Real-time RT-PCR analysis.

TRNzol (TIANGEN) was used to extract RNA from cells growing on tissue culture dishes. 500 ng RNA were used for following RT-PCR. The HiScript RT SuperMix kit (Vazyme) were used for generating cDNA according to the manufacturer's instructions. The qPCR was conducted in 384-well PCR microplates (Bio-Rad) in a CFX384 Real-Time System (Bio-Rad). Each well contained a total volume of 10 μl, including 4.2 μl cDNA (concentration ~2.5 ng/μl), 0.4 μl primers (10 μM), 5.0 μl Fast SYBR Green Master Mix (SYBR premix EX Taq, TaKaRa). The fluorescence was measured at the end of each cycle. Measured data was analyzed using the comparative CT method (ΔΔCT method). Mouse *Rpl13a* prime F: GGGCAGGTTCTGGTATTGGAT, R: GGCTCGGAAATGGTAGGGG; mouse *Mettl9* prime F: CTGGCAGCTCCAGAAGAAGA, R: CCACTTGCCACCTACGTTTT.

Cell proliferation and colony formation assay.

Cell proliferation was quantified using the cell counting Kit-8 (CCK8; APExBIO) according to the manufacturer's instruction. Cells were seeded in 96-well plates in triplicate at an initial density of 2000 cells per well. After being cultured for 24, 48, 72, and 96 hours, CCK-8 solution in 10 ul was added to each well, and cells were further incubated at 37 °C for 4 hours. Optical density (OD) value was measured spectrophotometrically at 450 nm wavelength.

Cells were plated in 6-well plates (10000 cells per well), and then cultured for 4 days. The overexpression cells were generated by infecting with BFP-Slc39a7 or mutants' virus. After 24 h, the BFP-positive cells were sorted by flow cytometry. For an assay of colony

formation, colonies were fixed with 4% paraformaldehyde for 20 minutes, stained with 0.1% crystal violet for 15 minutes, and then washed with phosphate-buffered saline (PBS) before measurement. The total colony area was measured by image J software. The relative area was calculated by total area divided by original cell number.

Tumor formation assay.

The C57B6/J mice and BALB/c nude mice were feeding under specific pathogen-free conditions in the experimental animal department of USTC. For the *in vivo* tumor formation assays, 1.5×10^5 RM-1 *Mettl9* KO cells (1#, 2#) or RM-1 wild type cells were subcutaneously injected into the C57B6/J mice (n = 5 for each group) and the BALB/c nude mouse (n = 6 for each group) at 8 weeks of age. After transplantation 8 days, the growth of the tumors was assessed every two days. The mice were sacrificed after a period of about 3 weeks, and the weights of subcutaneous tumors were measured.

Analysis of immune cell infiltrate in tumor microenvironment.

Preparation of single-cell suspension of mouse tumor tissues by mechanical grinding and collagenase digestion. For flow cytometry analysis, after blocking, cells were stained with the following mAbs: anti-CD45, anti-CD4, anti-CD8a, all from Biolegend. Samples were run on a BD flow cytometer and analyzed with the Flowjo Analysis Software (Beckman Coulter, Pasadena, CA). Cell populations were defined as follows: $CD45^+$ $CD4^+CD8^-$($CD4^+$ T cells); $CD45^+$ $CD4^-CD8^+$ ($CD8^+$ T cells).

IP assay and Mass spectrometry.

The 15 cm dish *Mettl9*-3×flag RM-1 cells extracts prepared with IP buffer (25 mM Tris-HCl pH 7.4, 150 mM NaCl, 1 mM EDTA,1% NP-40, 5% glycerol) remain on the ice for 30 minutes. Lysates were cleared by centrifugation at 12000 rpm for 10 minutes and incubated with anti-flag (anti-flag M2 Affinity Gel, Sigma) antibodies agarose. after 4 hours beads were then collected, and then the mass spectrum was performed. To select the METTL9-interacting proteins, the PEMs and Sum PEP Score was utilized to do a dot plot and top proteins closest to the upper right corner (PSMs > 2; log2(Sum PEP Score) > 2) was selected. For synthetic peptides methylation detection, after methylated by METTL9, the sample were sent to MS detection.

Expression and purification of recombinant proteins.

The appropriate pGEX-6P-1 constructs were transformed into the chemically competent BL21- Codon Plus (DE3)-RIPL (Agilent Technologies) E. coli strain. The bacteria were cultured until the OD reaching ~0.6, the mediums were transferred into 16 °C, 180 rpm incubator and protein expression was induced with 0.5 mM isopropyl β-D-1-thiogalactopyranoside (IPTG) followed by overnight growth. For GST-tagged proteins, bacteria were lysed in GST-Lysis Buffer (50 mM Tris-HCl, 600 mM NaCl, 1 mM EDTA, 1 mM DTT, 10 % Glycerol, Protease Inhibitor Cocktail, pH7.6) and then purified using GST-Sefinose(TM) Resin (BBI). GST-tagged proteins were eluted by 10 mM glutathione and GST was removed by enzyme ProScission.

***In vitro* methyltransferase assays.**

In vitro methyltransferase assays were performed using 30 ul reaction buffer (50 mM Tris-HCl pH 7.8, 50 mM KCl, 5 mM MgCl2) with 1 ul ^{3}H-AdoMet (Perkin Elmer, specifific activity = 55-85 Ci/mMole, 0.55 μCi/ul) or 1 mM unlabeled AdoMet for MS analysis of samples. For methylation of cell extract, reactions contained 80 μg substrates and 2 μg METTL9. For methylation of recombinant GST-tag proteins and 6×His Histone H3, reactions contained 2 μg substrates and 2 μg METTL9. Reactions were incubated at 37 °C for 1 h, and stopped by the addition of SDS-PAGE loading buffer. Proteins were separated by SDS-PAGE. For autoradiography analysis, the proteins were then transferred to PVDF membranes, stained with Ponceau S and exposed to XBT X-ray film (Carestream) for 30 days (cell extract) or 7 days (recombinant substrates). For methylation of synthetic peptides, reactions contained 5 μg substrates and 2 μg GST-METTL9 binding with GST-Sefinose (TM) Resin, GST-METTL9 was then removed by centrifugation. For autoradiography analysis, the peptides were directly loaded to NC membranes and exposed to XBT X-ray film (Carestream) for 7 days.

Fluorescence imaging of Zn^{2+} level.

Cell were cultured in the glass bottom micro-well dishes (Biosharp) for confocal microscopy imaging. Before ratiometric imaging with LSM 710 microscopy, 2 uM FluoZin-3 AM (Invitrogen) was add to dishes to stain cells for 1 hour and then was washed twice with PBS. For overexpression, the cells in 10 cm dish were infected with Slc39a7 wildtype or mutants in Plvx-IRES-BFP virus. After 24 h, the BFP-positive cells were sorted by flow cytometry. After cultured in the glass bottom micro-well dishes for 24 hours, the cells were

stained using FluoZin-3 AM (2 uM) for confocal microscopy imaging.

RNA-seq data processing and analysis.

The RM-1 cells of 1×10^7 WT and *Mettl9* KO were extracted with TRNzol (TIANGEN) for total RNA library preparation. The libraries are directly sequenced using next-generation sequencing technologies. After filtering, clean reads were mapped to the mouse reference genome. Mapping results were stored in BAM files. Total read counts at the gene level were summarized using feature-counts function in R environment, with the R package biomaRt for gene and transcript mapping. The differential expression genes were analyzed by DESeq2 with default settings using total read counts as input and the adjusted P value (p.adj) less than 0.05. Dotplot of differential genes of normalized expression matrix from DESeq2 analysis from GO pathway enrichment.

We performed gene set enrichment analysis (GSEA) using KEGG function of clusterProfiler package in RStudio. The RNA-seq data of non-targeting control (NC) and two individual *Slc39a7* siRNA in MDA-MB-231 cells are from the accession number GSE155437 from NCBI Gene Expression Omnibus (PMID: 33608508). Significant KEGG pathways with an enrichment score > 0.4 and a p-value <0.01 were identified. The R package ggplot2 was applied to visualize the results.

TCGA and GTEx data analysis.

We download the expression data of *METTL9* in PRAD, PAAD, LIHC combing GTEx normal sample from USCS Xena (http://xena.ucsc.edu), and make boxplot with significance difference index through ggplot in R environment.

We applied ESTIMATE computational method to calculate the immune score of different cancer samples from The Cancer Genome Atlas (TCGA) database. Correlation between several genes expression levels and immune scores were performed using the ‘rcorr’ function implemented in Hmisc R package v.4.5.0, adopting Pearson’s correlation method. Significant correlations (P<0.05) were plotted using the pheatmap R package v.1.0.12. Genes with no notable correlation show a coefficient equal to zero.

Statistical analysis.

All experimental operations were independently repeated for at least three times. Images was analyzed using Image J Software (National Institutes of Health, Bethesda, MD, USA). Statistical analysis was performed using GraphPad Prism Software 6 (GraphPad

Software, La Jolla, CA, USA). For data that had a normal distribution and homogeneity of variance, two-tailed Student's t test was performed to evaluate significant differences between two groups. P-values<0.05 were considered statistically significant.

SUPPLEMENTARY FIGURES

Figure S1

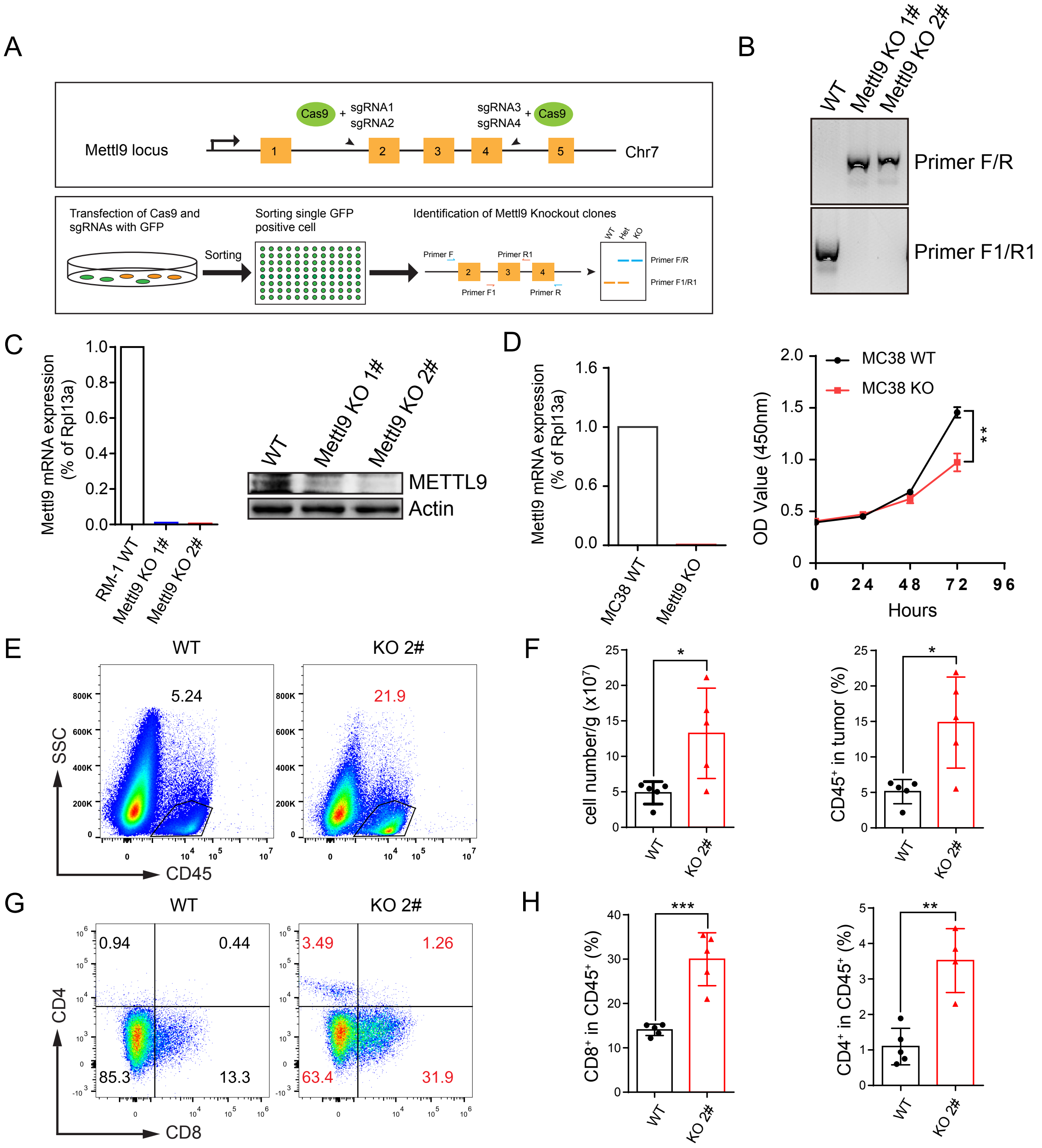

Figure S2

A

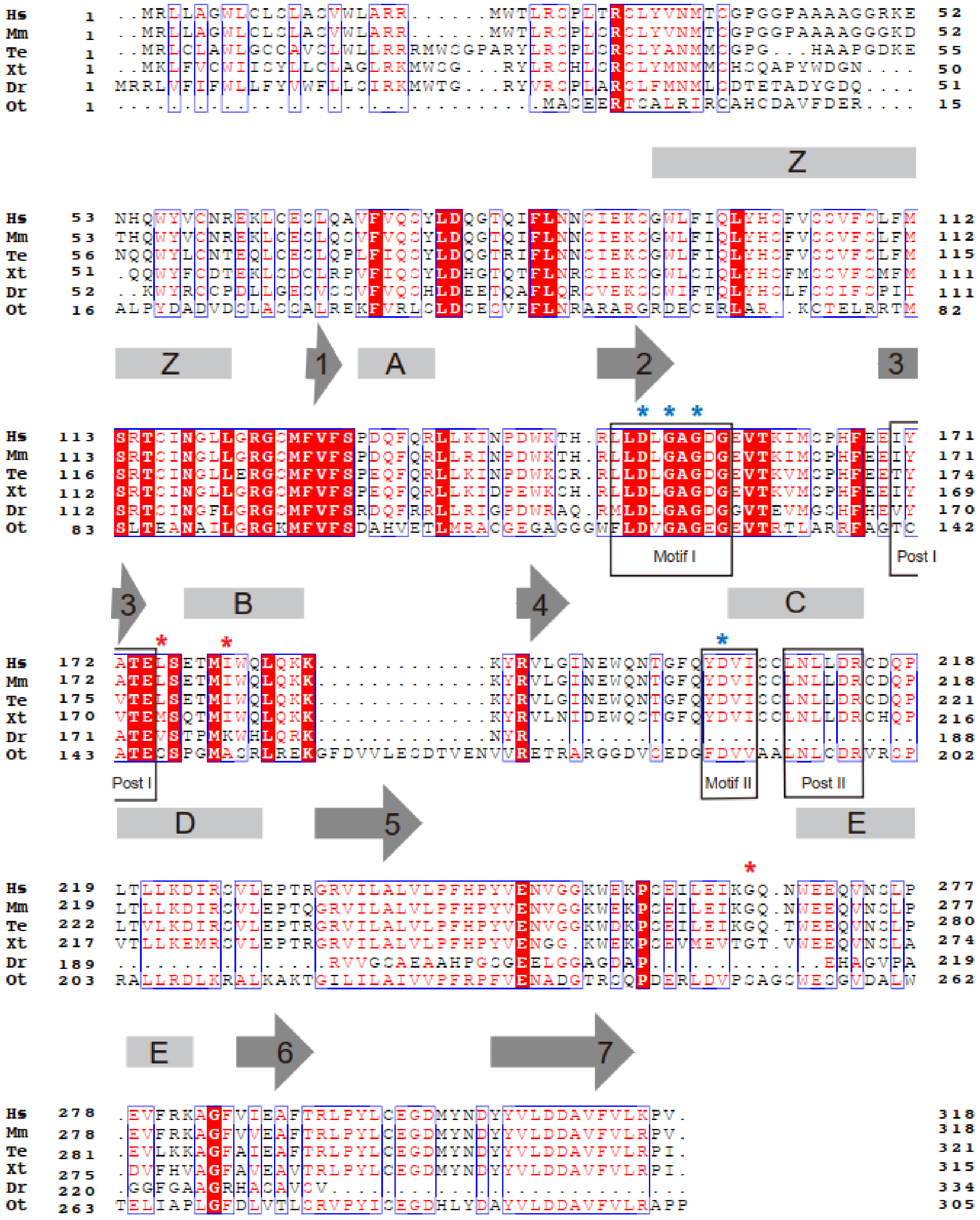

Figure S3

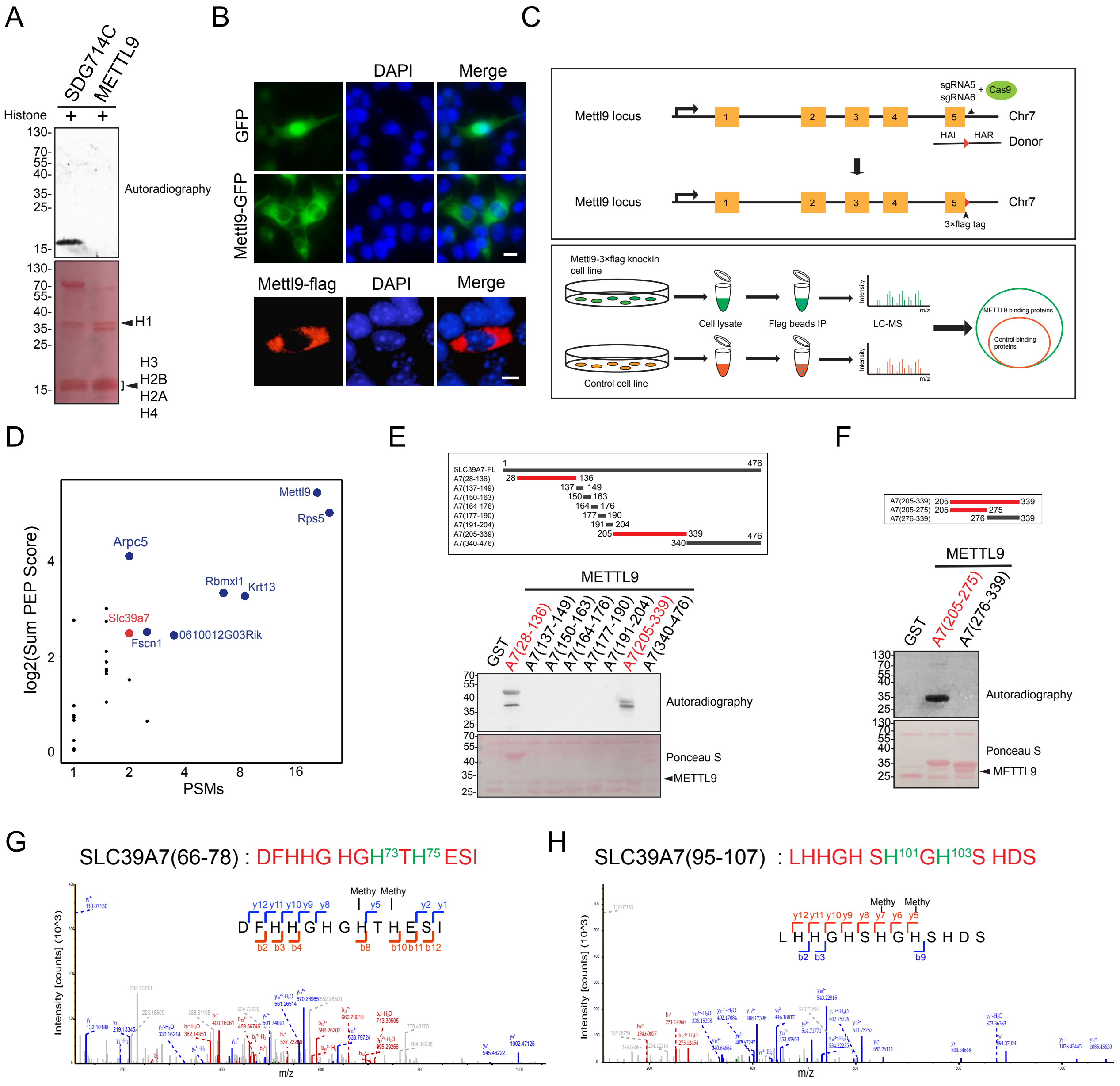
A
SDG714C
METTL9
Histone
Autoradiography
H1
H3
H2B
H2A
H4
B
DAPI
Merge
GFP
Mettl9-GFP
Mettl9-flag
C
Mettl9 locus
Chr7
Donor
HAL
HAR
sgRNA5
sgRNA6
Cas9
3×flag tag
Mettl9-3×flag knockin cell line
Cell lysate
Flag beads IP
LC-MS
Control cell line
METTL9 binding proteins
Control binding proteins
D
log2(Sum PEP Score)
PSMs
Mettl9
Rps5
Arpc5
Rbmxl1
Krt13
Slc39a7
Fscn1
0610012G03Rik
E
SLC39A7-FL
A7(28-136)
A7(137-149)
A7(150-163)
A7(164-176)
A7(177-190)
A7(191-204)
A7(205-339)
A7(340-476)
METTL9
GST
Autoradiography
Ponceau S
METTL9
F
A7(205-339)
A7(205-275)
A7(276-339)
METTL9
GST
Autoradiography
Ponceau S
METTL9
G
SLC39A7(66-78) : DFHHG HGH73TH75 ESI
Intensity [counts] (10^3)
m/z
Methy
H
SLC39A7(95-107) : LHHGH SH101GH103S HDS
Intensity [counts] (10^3)
m/z
Methy

FigureS4

A

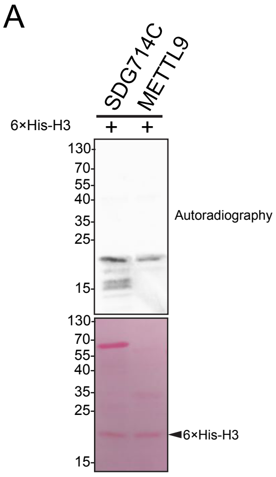
SDG714C
METTL9
6×His-H3
+
+
130
70
55
40
35
25
15
Autoradiography
130
70
55
40
35
25
15
6×His-H3

B

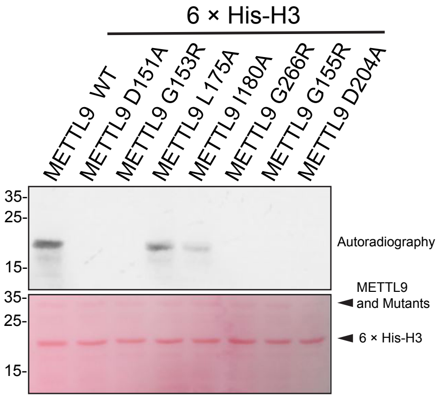
6 × His-H3
METTL9 WT
METTL9 D151A
METTL9 G153R
METTL9 L175A
METTL9 I180A
METTL9 G266R
METTL9 G155R
METTL9 D204A
35
25
15
Autoradiography
35
25
15
METTL9
and Mutants
6 × His-H3

Figure S5

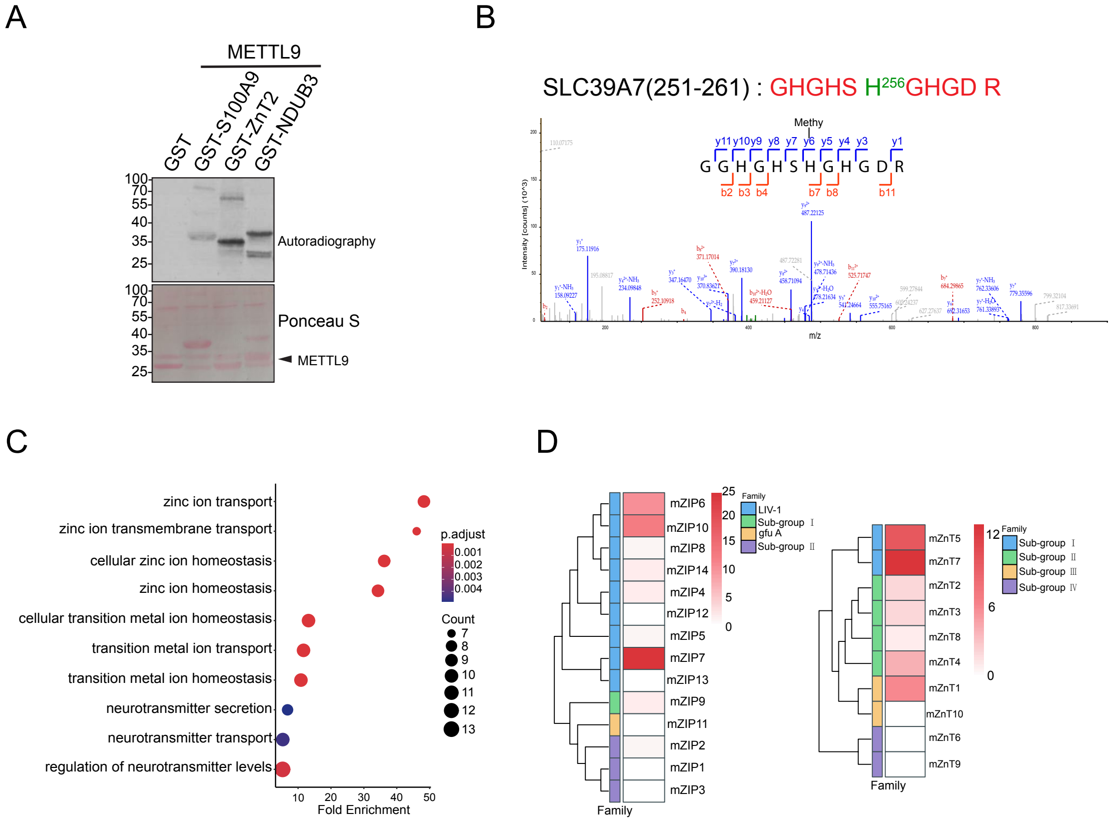
A
METTL9
GST
GST-S100A9
GST-ZnT2
GST-NDUB3
100
70
55
40
35
25
Autoradiography
Ponceau S
METTL9
B
SLC39A7(251-261) : GHGHS H256GHGD R
Methy
y11 y10 y9 y8 y7 y6 y5 y4 y3 y1
G G H G H S H G H G D R
b2 b3 b4 b7 b8 b11
m/z
C
zinc ion transport
zinc ion transmembrane transport
cellular zinc ion homeostasis
zinc ion homeostasis
cellular transition metal ion homeostasis
transition metal ion transport
transition metal ion homeostasis
neurotransmitter secretion
neurotransmitter transport
regulation of neurotransmitter levels
p.adjust
0.001
0.002
0.003
0.004
Count
7
8
9
10
11
12
13
10 20 30 40 50
Fold Enrichment
D
mZIP6
mZIP10
mZIP8
mZIP14
mZIP4
mZIP12
mZIP5
mZIP7
mZIP13
mZIP9
mZIP11
mZIP2
mZIP1
mZIP3
Family
25
20
15
10
5
0
Family
LIV-1
Sub-group Ⅰ
gfu A
Sub-group Ⅱ
mZnT5
mZnT7
mZnT2
mZnT3
mZnT8
mZnT4
mZnT1
mZnT10
mZnT6
mZnT9
Family
12
6
0
Family
Sub-group Ⅰ
Sub-group Ⅱ
Sub-group Ⅲ
Sub-group Ⅳ

Figure S6

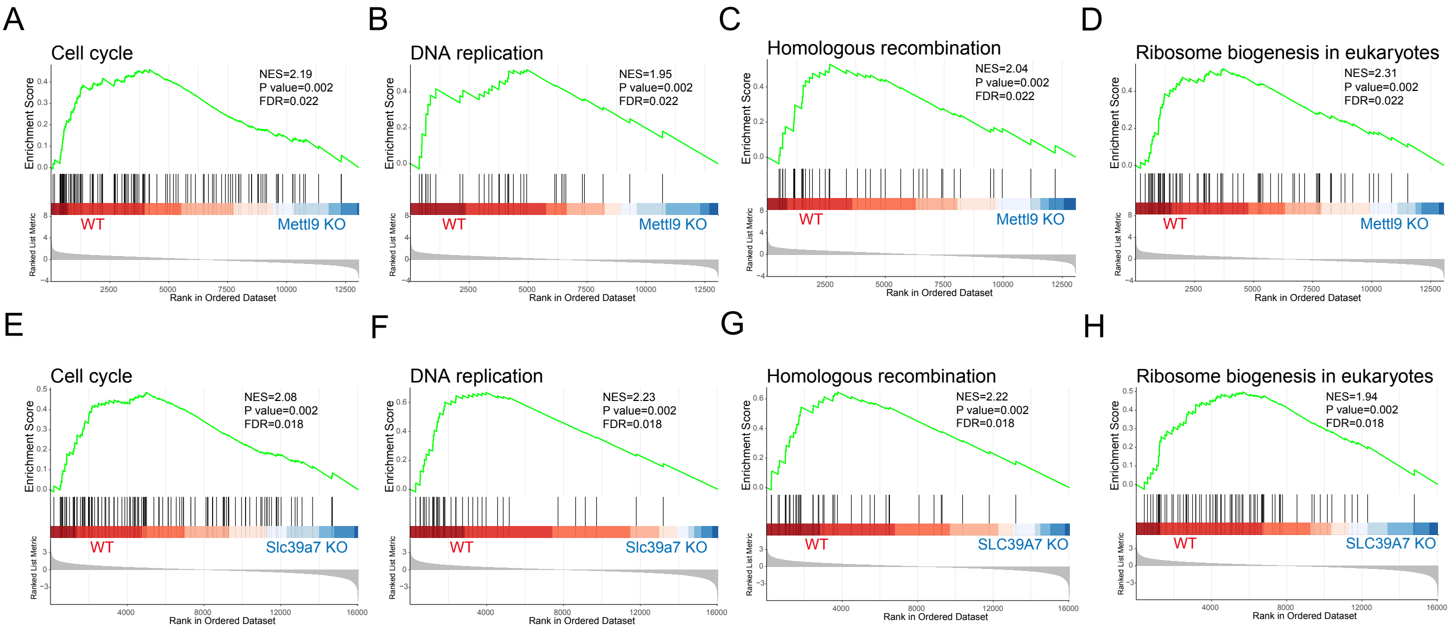

A
Cell cycle
NES=2.19
P value=0.002
FDR=0.022
WT
Mettl9 KO
B
DNA replication
NES=1.95
P value=0.002
FDR=0.022
WT
Mettl9 KO
C
Homologous recombination
NES=2.04
P value=0.002
FDR=0.022
WT
Mettl9 KO
D
Ribosome biogenesis in eukaryotes
NES=2.31
P value=0.002
FDR=0.022
WT
Mettl9 KO
E
Cell cycle
NES=2.08
P value=0.002
FDR=0.018
WT
Slc39a7 KO
F
DNA replication
NES=2.23
P value=0.002
FDR=0.018
WT
Slc39a7 KO
G
Homologous recombination
NES=2.22
P value=0.002
FDR=0.018
WT
SLC39A7 KO
H
Ribosome biogenesis in eukaryotes
NES=1.94
P value=0.002
FDR=0.018
WT
SLC39A7 KO
Enrichment Score
Ranked List Metric
Rank in Ordered Dataset

Figure S7

A

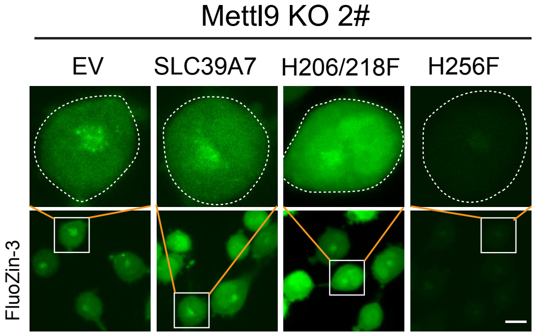

Supplement: Supplementary file 1 — Supplementary material 1 (PDF 7564 kb) [file 13238_2021_857_MOESM1_ESM.pdf]
